# Defining the Scope of Exposome Studies and Research Needs from a Multidisciplinary Perspective

**DOI:** 10.1021/acs.estlett.1c00648

**Published:** 2021-09-07

**Authors:** Pei Zhang, Christopher Carlsten, Romanas Chaleckis, Kati Hanhineva, Mengna Huang, Tomohiko Isobe, Ville M. Koistinen, Isabel Meister, Stefano Papazian, Kalliroi Sdougkou, Hongyu Xie, Jonathan W. Martin, Stephen M. Rappaport, Hiroshi Tsugawa, Douglas I. Walker, Tracey J. Woodruff, Robert O. Wright, Craig E. Wheelock

**Affiliations:** † Gunma University Initiative for Advanced Research (GIAR), Gunma University, Maebashi, Gunma 371-8511, Japan; ‡ Division of Physiological Chemistry 2, Department of Medical Biochemistry and Biophysics, Karolinska Institutet, Stockholm SE-171 77, Sweden; § Key Laboratory of Drug Quality Control and Pharmacovigilance (Ministry of Education), State Key Laboratory of Natural Medicine, China Pharmaceutical University, Nanjing 210009, P. R. China; ∥ Air Pollution Exposure Laboratory, Division of Respiratory Medicine, Department of Medicine, University of British Columbia, Vancouver, British Columbia V5Z 1M9, Canada; ⊥ Department of Life Technologies, Food Chemistry and Food Development Unit, University of Turku, Turku 20014, Finland; # Department of Biology and Biological Engineering, Chalmers University of Technology, Gothenburg SE-412 96, Sweden; ∇ Department of Clinical Nutrition and Public Health, University of Eastern Finland, Kuopio 70210, Finland; ○ Channing Division of Network Medicine, Brigham and Women’s Hospital and Harvard Medical School, Boston, Massachusetts 02115, United States; ◆ The Japan Environment and Children’s Study Programme Office, National Institute for Environmental Sciences, 16-2 Onogawa, Tsukuba, Ibaraki 305-8506, Japan; ¶ Science for Life Laboratory, Department of Environmental Science, Stockholm University, Stockholm SE-114 18, Sweden; 1 Division of Environmental Health Sciences, School of Public Health, University of California, Berkeley, California 94720-7360, United States; 2 RIKEN Center for Sustainable Resource Science, 1-7-22 Suehiro-cho, Tsurumi-ku, Yokohama, Kanagawa 230-0045, Japan; 3 RIKEN Center for Integrative Medical Sciences, 1-7-22 Suehiro-cho, Tsurumi-ku, Yokohama, Kanagawa 230-0045, Japan; 4 Department of Biotechnology and Life Science, Tokyo University of Agriculture and Technology, 2-24-16 Nakamachi, Koganei, Tokyo 184-8588 Japan; 5 Graduate School of Medical life Science, Yokohama City University, 1-7-22 Suehiro-cho, Tsurumi-ku, Yokohama 230-0045, Japan; 6 Department of Environmental Medicine and Public Health, Icahn School of Medicine at Mount Sinai, New York, New York10029-5674, United States; 7 Program on Reproductive Health and the Environment, University of California San Francisco, San Francisco, California 94143, United States; 8 Department of Respiratory Medicine and Allergy, Karolinska University Hospital, Stockholm SE-141-86, Sweden

## Abstract

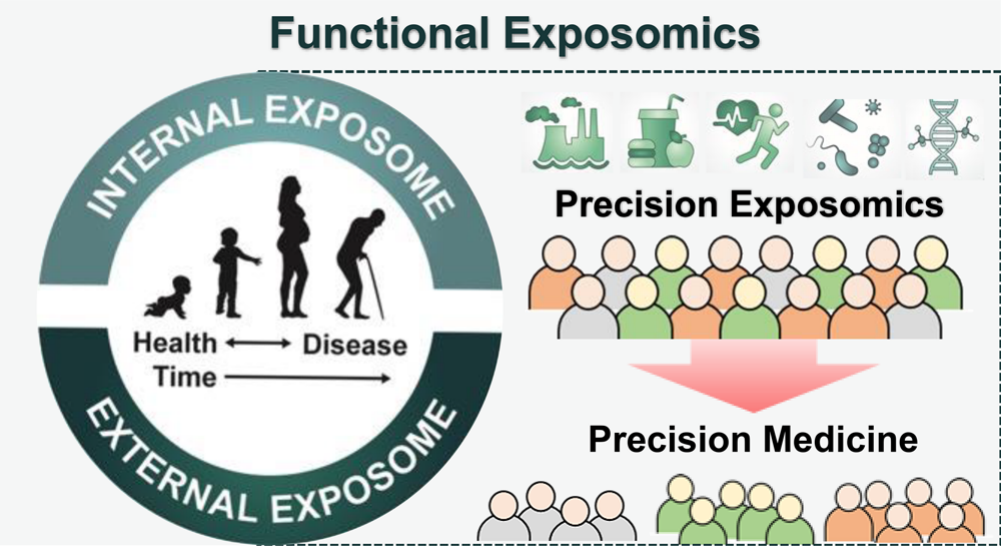

The concept of the exposome was introduced over 15 years ago to reflect the important role that the environment exerts on health and disease. While originally viewed as a call-to-arms to develop more comprehensive exposure assessment methods applicable at the individual level and throughout the life course, the scope of the exposome has now expanded to include the associated biological response. In order to explore these concepts, a workshop was hosted by the Gunma University Initiative for Advanced Research (GIAR, Japan) to discuss the scope of exposomics from an international and multidisciplinary perspective. This Global Perspective is a summary of the discussions with emphasis on (1) top-down, bottom-up, and functional approaches to exposomics, (2) the need for integration and standardization of LC- and GC-based high-resolution mass spectrometry methods for untargeted exposome analyses, (3) the design of an exposomics study, (4) the requirement for open science workflows including mass spectral libraries and public databases, (5) the necessity for large investments in mass spectrometry infrastructure in order to sequence the exposome, and (6) the role of the exposome in precision medicine and nutrition to create personalized environmental exposure profiles. Recommendations are made on key issues to encourage continued advancement and cooperation in exposomics.

## Introduction

As early as the 18th century, it was demonstrated that environmental exposures increase risks of chronic human disease.^1^ Public awareness for this idea grew in the 1950s when causal links were reported between smoking and lung cancer.^2^ Soon afterward, Rachel Carson’s “Silent Spring” raised concerns about the adverse health consequences of synthetic organic chemical exposures,^3^ thus motivating establishment of the U.S. Environmental Protection Agency (EPA) and many new funding avenues for research into the occurrence and health consequences of environmental contaminant exposures. The *Environmental Science and Technology* (*ES&T*) journal has communicated such research to an international audience since 1969.^4^ However, while myriad environmental exposures have since been discovered—of which only a few hundred have been studied for disease risks—the underlying methods of exposure assessment and environmental epidemiology have remained remarkably static. There has been a shift from occupational studies of workplace exposures to population-based environmental studies to research on multiple factors (e.g., age specific vulnerability) and low dose effects. However, epidemiology studies still tend to focus on single chemicals or a handful of related chemicals (e.g., phthalates), rather than real-world mixtures.

Environmental monitoring and human biomonitoring have historically been hypothesis driven, addressing environmental contaminants one at a time. Today, thousands of targeted analytical methods exist for the accurate measurement of contaminants in biofluids, food, air, drinking water, and soil. Nonetheless, each method tends to be applied to only a small number of chemicals having similar properties or structures.^5−7^ The health risks of single chemical exposures, or the sum of chemicals within a related chemical class (e.g., polychlorinated dioxins^8^), are estimated by comparison of measured levels to dose–response relationships derived from animal studies, which are also conducted one chemical at a time. In environmental epidemiology, associations between single chemicals or chemical mixtures are investigated over a wide range of exposures to strengthen causal inference.^9,10^ Although these chemical-by-chemical approaches are valuable for confirming *a priori* hypotheses, they are unsuited for discovering health effects that arise from the vast majority of still unknown exposures not yet measured in environmental media or biospecimens. The exposome concept addresses this issue, in part through more comprehensive or unbiased exposure measurements; however, because the number of chemicals we can measure is now so large, studies need to be designed differently to avoid promoting false positive findings.

The practical limitations of targeted exposure-effect studies are obvious. A recent review of chemicals in commerce identified more than 350,000 chemicals that are registered for production and use.^11^ The number of synthetic chemicals that comprise real-world exposures is even greater because chemicals in commerce may be complex mixtures or contain isomers and impurities, and many are transformed in humans or by microbes in the body (i.e., the microbiome) or in the environment (e.g., methylmercury) to a multitude of degradation products. Beyond exposure to commercial chemicals, we breathe, drink, and eat complex mixtures of pollutants from anthropogenic emissions to air and water, and even the cooking of food introduces potentially carcinogenic byproducts.^12^ In addition, the greatest intake of the chemical exposome is through diet, a massive contributor to the exogenous biochemical load, consisting of thousands of natural or anthropogenic chemicals that impact our endogenous metabolism and fine-tune risk of diseases.^13−15^ Natural substances in food, air, and water may affect health directly^16,17^ but may also interact with effects posed by environmental contaminants.^18^ In addition, environmental exposures to nonchemical stressors, including noise, light, social, and socioeconomic factors and green space and climate, affect biological responses and may also interact with chemical exposures.^19−21^ Additional complications arise from variability in exposures and effects due to changing locations, age, lifestyle, diet, sex, ethnicity, and health status.^22−24^

The complex milieu of real-world exposures highlights the limitations of targeted methods for exploring causes of disease. Moreover, experimental and observational studies evaluating adverse effects typically focus on doses or exposures to a single chemical, which is quite different from those presented by mixtures,^25,26^ and unmeasured coexposures can confound results of targeted studies.^27,28^ Among the largest systematic human biomonitoring programs in North America and Europe (NHANES^29^ and HBM4 EU,^30^ respectively), only ∼300 chemicals are routinely analyzed by targeted methods in human biofluids. Moreover, owing to limitations of the sample volume needed, this list of chemicals has never been measured collectively in a single individual. Accordingly, cumulative environmental chemical exposures and their effects are still poorly understood in population studies.

The “exposome” was first proposed as a research priority ∼15 years ago to recognize the important roles that environment plays in cancer (and by extension other chronic diseases).^31^ The concept was motivated by recognition that the genome alone explained only a small proportion of the population variance of chronic disease in developed countries.^32,33^ The exposome was intended to represent everything that the genome did not, and if it could be adequately characterized in sufficient numbers of people throughout their life course, it promises to reveal nongenetic causes of disease and gene–environment interactions (i.e., genome × exposome interactions).^34−37^ Although there has been a call to “sequence the exposome”,^38^ there is currently no agreement as to how this could be accomplished.^39^ Acquiring data for all exposures is one obstacle, but linking such data to health information brings additional challenges. Nonetheless, the number of publications using the term exposome is increasing exponentially, and granting agencies are beginning to provide support for large exposome studies.^40−43^

In order to discuss the exposome and its collective challenges, the second International Exposome Symposium of the Gunma University Initiative for Advanced Research (GIAR) was organized. Speakers were invited from Europe, North America, and Japan with a range of expertise in the areas of medicine, epidemiology, data science, environmental toxicology, analytical chemistry, and food science (Figure S1). This article summarizes and integrates the presentations and subsequent discussions with respect to (1) defining terminology and the scope of the exposome, (2) considerations in designing exposome studies, (3) characterizing the exposome via high-resolution mass spectrometry, (4) developing computational strategies for exposomics data, (5) producing databases for exposures and exposure-disease relationships, and (6) predicting roles of exposomics in precision medicine and precision nutrition.

## Scope of the Exposome

For the exposome to achieve widespread adoption, multiple disciplines will need to work together, including environmental scientists, social scientists, analytical chemists, molecular biologists, toxicologists, epidemiologists, and physicians. Unlike DNA sequencing, where a common technology can accurately and reproducibly characterize an individual’s genome, the exposome is highly dynamic in time and space and requires a range of tools to measure it. Furthermore, effects of exposure are complicated by the quantal nature of dose–response relationships within a population, where individual responses vary with exposure history, age, age at exposure, genetics, and coexposures. As a result, an individual’s exposome tends to be defined by the analytical and methodological approaches used in a given study.^20^

**Figure 1:**
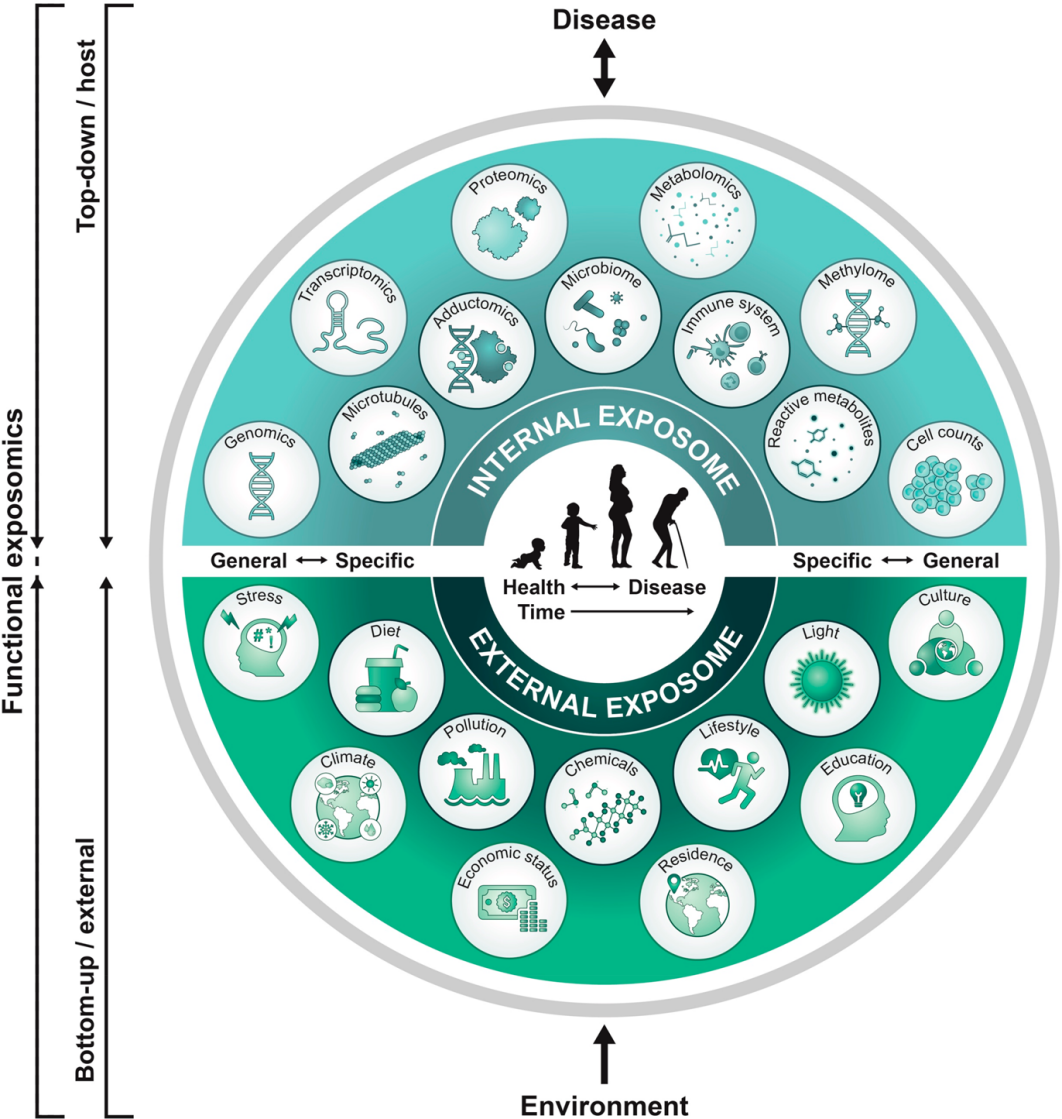
Functional exposomics approach to study the exposome. In the top-down approach, molecular epidemiology studies focus on exposure (e.g., small molecule biomarkers of exogenous compounds, protein adducts, reactive metabolites) and biological response profiles (e.g., metabolomics, gene expression, methylation) within the host using biospecimens. This approach can generate hypotheses regarding exposure–disease and exposure–response relationships but does not necessarily capture direct measures of exposure. In the bottom-up approach, comprehensive data on environmental exposures are collected through surveys, sensors, or trace analytical chemistry in environmental samples (external exposures) or in biospecimens. This can generate hypotheses on effects but does not necessarily investigate the effect. We propose that a functional exposomics study bridges these two approaches and consists of the biologically active exposures present in an individual and specifically examines associations between environmental exposure and biological effect.

Since the original conceptualization of the exposome, the definition has evolved to incorporate omics technologies that can be harnessed to characterize exposures.^44^ The exposome was originally envisioned in 2005 from Dr. Christopher Wild’s perspective as a cancer epidemiologist as “. . .encompassing life-course environmental exposures from the prenatal period onwards”.^31^ Here, the emphasis was on improving exposure characterization to find causes of disease that moved beyond traditional approaches of individual targeted exposures. A few years later, Rappaport and Smith considered two exposomics approaches for finding causal human exposures.^45,46^ The bottom-up approach that would characterize chemicals in environmental media (e.g., air, water, diet, the built environment) and the top-down approach that would focus on chemicals measured in biospecimens (Figure 1).

More recently, an expansion of the bottom-up definition has been made to include combinations of external exposure to chemical, physical, psychological, and social factors. As comprehensive understanding of bottom-up exposures increases, new and more sophisticated hypotheses can be developed regarding potential health consequences. Concurrent to advances in analytical chemistry, methods to measure the external exposome are also expanding. Geospatial methods to measure indirect exposure include satellite remote sensing to measure air pollution, human activities, and green space among other end points.^47^ Census data can be mined for neighborhood characteristics. Public databases contain extensive information including crime, infections, environmental measures, and pesticide application rates, as well as geospatial data that can be linked to home address, school, or work.^48−50^ Personal air samplers (passive and active)^51−55^ can be worn to monitor bottom-up exposures in the near-field environment, and smartphone apps or other wearable devices, such as smart watches, increasingly can be used to “crowd source” measures of noise, activity, and social factors and link them to physiologic measures.^56^ Recent work has even detailed an integrated pipeline to analyze a full complement of biotic and abiotic exposures in the personal airborne exposome.^57^ A great deal of potential for understanding social networks can be derived from social media platforms, rendering measures of the exposome even more robust and helping to identify sources of variability in the detected endogenous chemicals.

In contrast, the top-down strategy for finding causes of disease relies on samples of human biospecimens to simultaneously investigate exposures originating both inside and outside the body. By employing untargeted omics to compare exposomes in biospecimens from diseased and healthy subjects, it is now possible to discover potentially causal chromatographic features and use them to generate hypotheses for follow-up studies that confirm their chemical identities, identify sources of exposure, and establish exposure–response curves.^36,58−60^ Since blood is the most common biospecimen that is archived in prospective-cohort studies, this top-down strategy led to the concept of the “blood exposome” and broadened the universe of exposures to include pollutants, diet, drugs, and endogenous chemicals.^17^ More recent definitions of the exposome have been formulated to include the collection of other omics methods—metabolomics, metallomics, adductomics, proteomics, and metagenomics—that can characterize exposures^61^ and the molecular changes associated with exposures.^60,62−66^ Critical to this latter definition is the notion that a cumulative biological effect can be used to evaluate overall exposure and allostatic load.^60^ Incorporating biological responses within a top-down framework enables an understanding of how exposures exert stress on host homeostasis, while potentially revealing causal pathways and mechanisms underlying exposure–disease relationships. Recent applications of this approach that combine exposure monitoring with biological response, where multiple exposures are assessed in individuals and compared with phenotype or omics profiles, have provided novel insight into the role of environment in disease risk.^36^ This is exemplified by linking dietary exposure with various clinical outcomes and disease risk in an epidemiological setting using metabolomics-based approaches.^67,68^ However, while allostatic load can be estimated, the root causes of these stressors and the role of exposure timing, route, or source cannot be captured by a top-down approach and may even be absent from the analysis, limiting the ability to develop interventions.

Full characterization of causal exposome features and subsequent interventions requires knowledge of exposure sources. This is relatively simple when exposures are measured bottom-up in external media, such as air, water, or food (external exposome), but can be more complicated when measurements are made top-down in biofluids (internal exposome^66^). Herein, we propose to further distinguish measurable components of both approaches for characterizing the exposome. Accordingly, the exposome can be divided into the following four categories (Figure 1): Bottom-up: (i) general external exposures including the built environment, climate, air pollution, social stressors, socioeconomic factors, etc. and (ii) specific external exposures such as chemical contaminants, diet, occupational exposures, or medication. Top-down: (iii) internalized exogenous exposures that comprise the fraction of non-nutrient environmental molecules that have entered the organism and (iv) endogenous nutrient exposures, including gut microbiota and their associated metabolites, that arose either directly from the diet or are products of endogenous metabolism reflecting the exposure (e.g., lipid peroxidation products from oxidative stress, etc.). A combination of these four categories provides a framework for linking external exposure to internal dose, biological response. and adverse health outcomes, thus defining an individual’s functional exposome.

While bottom-up and top-down definitions of the exposome can aid in study design, the greatest potential in the application of exposomic approaches lies in integrating the top-down approach with the bottom-up approach. This approach, which we define here as functional exposomics, has a greater scope that enables synergy by combining internal measures of exposure and biological response with measures of the external environment in order to identify exposure sources, the source of the biological response, and to better establish disease causality.^69^ For example, untargeted high-resolution mass spectrometry (HRMS) approaches may demonstrate that the small molecule chemical profiles in biofluids are central in linking external exposure (i.e., measured as environmental chemicals of concern in food) to internal dose (i.e., exposure biomarkers), biological response (measured as alterations in endogenous metabolic pathways), and disease (through shifts in metabolism linked to disease pathogenesis). This information can be linked to source of exposure (e.g., air, water, diet) to provide a complete understanding of the relationship between environment/exposome and health or disease outcome.^67,68,70^ This has been demonstrated by recent studies linking occupational exposures to a chlorinated volatile organic solvent with alterations in both the exogenous and endogenous metabolome,^71^ metabolomic phenotypes of exposure to common traffic-related air pollutants, and metabolite changes detected in individuals decades after an initial exposure occurred.^71−74^ Incorporating measures of additional omics layers within the exposome enables a systems–biology framework to study the effects of exposures, providing, for the first time, the comprehensive characterization needed to elucidate potential toxicological mechanisms at the population level.^75^ A recent report proposed eight hallmarks of environmental insult that jointly lay the foundation for the health consequences of environmental exposures.^76^

The current work focuses on the role of small molecules in the exposome of the type that are generally analyzed in untargeted metabolomics-based efforts using HRMS. However, the other “omic” approaches also play an important role in exposomic science. In particular, integrative “omics” offers the potential to provide global patterns coupled to metabolic and physiological dysregulations and capture the biological complexity associated with exogenous exposures. One omic technology that has been explored in detail is adductomics,^77−79^ which has been proven useful for identifying exposures to exogenous compounds and in newborn blood spots.^64^ While beyond the scope of this work, interested readers are encouraged to explore examples of the applications of proteomics,^80^ genomics,^81^ and epigenetics^82^ to investigating the exposome. It is expected that these technologies will continue to contribute to our understanding of the health effects of environmental exposure.^83^

The necessity of exposomics will be further amplified by the consequences of climate change, with multiple ramifications for the environment and the ensuing effects upon human health.^84,85^ Accordingly, exposomics should be considered an important component of climate change research. In addition, although all these approaches are largely discussed from a human perspective, they can be equivalently applied to other organisms. For example, the polar bear blood exposome has been examined to identify the specific chemicals that lead to thyroid disruption.^86^ Other laboratory animal models are being used to simulate human exposome conditions, such as to combinations of dietary and occupational exposures, but also to understand aquatic exposomes downstream of municipal wastewater,^87^ as well as for real-world applications due to tire rubber-derived exposure.^88^ Nonhuman exposome studies will be important for the protection of wildlife and ecosystems as recently demonstrated for coho salmon^88^ and will also protect humans under the one health paradigm.^89,90^

## Considerations in Exposome Study Design

Exposomics is a nascent field with unique data requirements, thus existing cohorts and ongoing studies may not be optimal for exposome studies.^91^ For example, many existing large-scale metabolomics studies were designed to explore associations between nutrient metabolites and health outcomes, and general demographic and disease-specific clinical parameters were collected with this sole intention. Data on environmental exposures, or exposure biomarkers, and broader health conditions are often lacking, and biological samples may not be of sufficient quality or quantity for comprehensive and optimal exposomic analyses. An atlas or reference exposome study has not yet been conducted but is sorely needed. Such a study would be analogous to efforts in genomics to haplotype different populations around the world (i.e., the Hapmap). Because the exposome will vary by culture, geography, time (i.e., the 1990s are different from the 2020s) and life stage (an infant is different from an adult), mapping a reference exposome will require significant resources, similar to the Hapmap project. However, the potential benefit to researchers is enormous because it will enable us to better understand the role of culture, geography, life stage, and time in predicting the exposome and improve our interpretation of results enabling better causal inference. Such a large-scale global project should be a major priority for researchers and will require substantial resources and collaboration. With respect to the more typical exposome study, we propose a suite of guidelines that are described in the Supporting Information.

## Measuring the Chemical Exposome by Mass Spectrometry

There are multiple approaches for data acquisition that should be considered in comprehensive exposome studies, including questionnaires, mobile sensors for air quality^92^ and noise,^93^ UV exposure,^94^ and physical activity,^95,96^ as well as smartphone apps to assist with acquisition of dietary data.^97^ Nevertheless, given the great potential for making molecular links between bottom-up and top-down studies, the current discussion focuses on the application of mass spectrometry for acquiring the small molecule chemical exposome, with an emphasis on HRMS acquisition and untargeted data analysis.

Considering the complexity of the chemical exposome, it is unlikely that there will soon be a single untargeted method capable of capturing the full range of small molecules that are present in biofluids or environmental samples.^98^ In a review of the human blood exposome by Rappaport et al.,^17^ the concentrations of 94 known pollutants, 49 drugs, 195 food chemicals, and 1223 endogenous chemicals spanned 11 orders of magnitude in blood, from 160 fM to 140 mM.^17^ Considering that modern mass spectrometers are at best linear over 5–6 orders of magnitude, several types of unbiased sample extractions and untargeted analytical methods will be required to achieve detection and semiquantification for a comprehensive profile of small molecules in human blood.^98^

**Figure 2:**
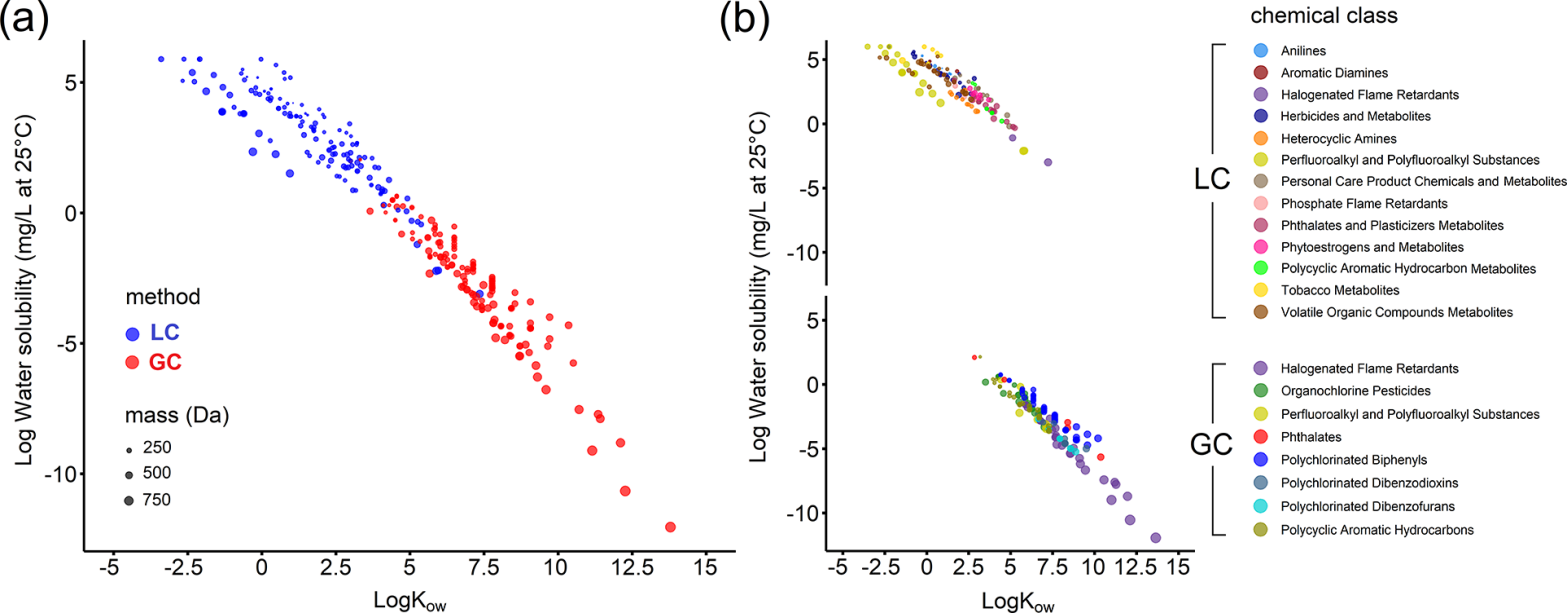
Full coverage of the chemical exposome will require multiple instrumental approaches, as shown by the chemical space of 299 internal exogenous analytes routinely targeted in large population biomonitoring studies in blood or urine. a) Measurement of the analytes will require a mixture of LC- and GC-based approaches that are b) dependent upon the analyte class. Analytes were selected from the National Health and Nutrition Examination Survey (1999–2016, latest update in 2019), Centers for Disease Control and Prevention (USA), and HBM4 EU (European Environment Agency, and European Commission, latest update in 2018). Water solubility and K_ow_ values are estimated from EPA EPI Suite software and span 18 orders of magnitude for water solubility and 15 orders of magnitude for K_ow_. Estimations of the K_ow_ values for the anionic perfluoroalkyl acids included in the class of perfluoroalkyl and polyfluoroalkyl substances are from Hidalgo and Mora-Diez.^163^

Untargeted high-resolution metabolomics analysis, based on liquid chromatography (LC) and HRMS has been proposed for “sequencing the exposome”,^38^ but clearly, a wider range of instrumental approaches will be required. This can be exemplified by considering the wide range of organic contaminants routinely monitored in human blood by target methods (Figure 2). These analytes span approximately 18 orders of magnitude in water solubility and 15 orders of magnitude in octanol–water partition coefficients. Only half of these analytes are relatively water soluble and have polar functional groups that can be ionized under atmospheric pressure, making them amenable to LC-HRMS workflows. The other half are relatively nonpolar and semivolatile and are best analyzed by gas chromatography (GC)-HRMS workflows. Untargeted GC-HRMS is therefore becoming increasingly popular as a complement to LC-HRMS, which together enable a more comprehensive coverage of the small molecule exposome.^99−102^ Furthermore, considering that prominent hydrophobic organic contaminants are preferably analyzed by GC, and their biotransformation products are only detectable by LC,^103^ the combination of both instrumental approaches in untargeted modes could simultaneously reveal exposure sources and individual variation in biotransformation capacity. For truly comprehensive exposure, trace metals in blood should be analyzed by another method such as inductively coupled plasma mass spectrometry (ICP-MS).^104^

HRMS technologies are an essential component for characterizing the human exposome because of their inherent sensitivity, dynamic range, and high-frequency full scanning with high mass accuracy.^105,106^ The present capabilities of these instruments to collect full scan MS^1^ and parallel MS^2^ data, by data-dependent or data-independent approaches, opens possibilities to identify known substances through formula assignment and spectral library matching, with the additional possibility to annotate and structurally characterize complex “molecular dark matter”, which constitutes the majority of all HRMS signals in typical samples.^107−112^ In addition, the advent of ion mobility technologies can provide further resolving power for untargeted approaches.^113−116^ However, critical challenges remain. As described by Rappaport et al.,^17^ the abundance of environmental chemicals in biological samples is, on average, 3 orders of magnitude lower than endogenous metabolites, drugs, or dietary components. Thus, going beyond the metabolome into the exposome necessitates higher sensitivity instruments or methods. Unbiased sample preparation methods should be further developed to comprehensively concentrate the trace small molecule exposome, while minimizing matrix suppression by highly abundant endogenous metabolites (e.g., phospholipids). Although untargeted HRMS data acquisition strategies are not inherently quantitative (i.e., few internal standards, lack of external calibration curves for most analytes), a strategy has been developed and validated that allows retrospective quantification of analytes discovered in untargeted exposome studies. The so-called “reference standardization protocol” makes use of concurrently analyzed pooled reference samples and was shown to be comparable to surrogate standardization or internal standardization.^117,118^

Sample throughput in HRMS approaches remains an obstacle to large exposome studies, and this is compounded here by our recommendations that multiple mass spectrometry methods should be applied to individual samples. For example, the Japan Environment and Children’s Study (JECS)^119−121^ cohort has hundreds of thousands of individual samples, which may not be feasible for the current state of HRMS without many dedicated instruments working in parallel. As a reference point, JECS is currently analyzing 5000–20,000 samples by mass spectrometry per year for heavy metals, PFASs, and phthalate metabolites, as well as some insecticides; however, this throughput is not currently feasible for brominated/phosphorus flame retardants, dioxins, and some pesticides that require complex sample preparations. Targeted LC-MS/MS methods (i.e., with MRM transitions) are becoming more comprehensive and can be adapted to increase throughput in exposomics studies. For example, a recent platform was able to analyze 1000 metabolites or exposome-related compounds using a triple quadrupole linear ion trap instrument.^63^ Conversely, it is not always necessary to analyze thousands of chemicals. Attempts have been made to prioritize chemicals for exposome-based studies, enabling the development of targeted quantitative methods suitable for low-abundant molecules that are difficult to measure with screening approaches.^122^

While there is a necessity to increase mass spectrometry throughput, there is a concomitant need to increase institutional investment in facilities for exposome studies at a scale that approaches the investments in genome sequencing technologies. In order to actionize the exposome on the scale of the Human Genome Project, extensive support will be required from funders. Concurrent with this investment, there is a requisite need for method standardization and data harmonization. A good example of these efforts is the EPA’s Non-Targeted Analysis Collaborative Trial (ENTACT), which included nearly 30 laboratories to characterize 10 synthetic chemical mixtures, three standardized media (human serum, house dust, and silicone band) extracts, and thousands of individual substances using GC- and LC-HRMS approaches.^123^ While it is not realistic or even desirable for every laboratory to employ identical methodologies, common retention indexing in LC and GC approaches, as well as common reference materials (e.g., NIST SRM1950 for plasma) could assist in producing data that can be combined across independent laboratories.^124^ For example, the concentration of small molecules could be normalized based on their fold difference relative to human plasma (NIST SRM1950) or semiquantified against other common reference materials using reference standardization.^117^

A number of existing resources support MS-based untargeted small molecule profiling and identification, including open-access software for data preprocessing (e.g., MS-DIAL,^125^ MZmine,^126^ XCMS^127^) and compound databases and spectral libraries (e.g., Massbank,^128^ GNPS,^129^ HMDB,^130^ T3DB,^131^ and PubChemLite^132,133^), as well as resources dedicated to cover the exposome and its associated metabolism (e.g., Exposome-Explorer,^134^ NORMAN (https://www.norman-network.com/), and CECscreen^135^). Confidence in annotations can be strengthened by using a standardized retention indexing system. For GC, a robust system already exists (Kovats retention index, using a series of alkanes), but for LC, there is currently no widely accepted method for any mode of chromatography. Efforts have been made to establish a similar strategy for LC-MS with a series of 2-dimethylaminoethylamine (DMED)-labeled fatty acids.^136^ Moreover, new approaches based on drift time in ion-mobility separation (i.e., based on collision cross section) in modern hybrid mass spectrometers show alternative promise.^137,138^ Software for data analysis that can accommodate both LC- and GC-HRMS chromatograms, while deconvoluting MS^2^ data, must continue to be optimized and validated.^139^ Ideally, the software and supporting spectral libraries should be vendor neutral (e.g., mzXML) to enable experimental replication and to support open science activities under FAIR data management principles (findability, accessibility, interoperability, and reusability). A bottleneck in exposome studies is the structural characterization of hundreds or thousands of important, yet unknown, molecular features in human or environmental samples, but powerful open science tools are increasingly available that combine untargeted MS^1^ and MS^2^ data for high-throughput structural characterization by molecular networking.^140^ These resources and approaches, if commonly adopted, would open the possibility to combine several small exposome studies into larger and more powerful metastudies.

## Working with Exposome Data

### A Call to Create a Comprehensive Exposome Database

There have been a number of initial efforts in exposome database construction.^141,142^ The EPA CompTox Chemical Dashboard, in particular, is an excellent source that currently contains 882,000 chemicals as of December 2020.^143^ In addition, the NORMAN network (http://www.norman-network.net/) contains extensive information on emerging substances in the environment, and the recent COlleCtion of Open Natural prodUcTs (COCONUT) database provides over 400,000 unique natural products.^144^ However, the majority of databases focus on the parent structures of exposures and lack information on the biological and microbiota transformation products, which can serve as internal biomarkers of both exposures and biological processes. Moreover, the dark matter of the metabolome (known unknowns^112^) in the existing databases is largely missing. Incorporating known unknowns into future methods or data analysis workflows can be done by sharing suspect lists (e.g., 10.5281/zenodo.2656745)^145^ with associated confidence levels for compound annotation^146^ and even proposed structures. Likewise, the current repositories for food-borne compounds still lack spectral information essential for identification purposes, and in general, the biochemical diversity in foods remains relatively poorly catalogued.^147^ In the future, this can be vastly improved by retention index reporting and associated MS^2^ spectra. In current databases, various nomenclature methods are used including IUPAC Name, InChI, InChIKey, SMILES, CHEBI, and CAS Number. There should be a common nomenclature and ontology dedicated to exposome chemicals, and InChIKey might be the best choice because it facilitates the search and share of chemical information and is commonly used in most databases due to its fixed length and format. In order to make it comprehensive, an exposome database should also include organometallic compounds that are not routinely analyzed by HRMS-based approaches. With respect to the analysis, interpretation, and reporting of exposomic data, we provide additional discussion and details in the Supporting Information.

## Precision Diagnostics

A potential future application of exposomics is in the area of diagnostic tools. The relative transiency of metabolic changes across individuals may be limiting in this regard, but for chronic illnesses (i.e., cancer, liver disease), disease specific signatures have already been described.^148,149^ A potentially overlooked area of research is nontraditional biological matrices—such as skin, hair, or toenails. Medicine has traditionally focused on blood and urine as the major biosamples for routine medical monitoring. However, tissues with slow growth rates may offer complementary findings that reflect integrated changes in metabolism over time.^150−152^ While exogenous chemicals can contaminate these tissues, focusing on endogenous metabolites (e.g., cotinine, cortisol) has been validated in targeted assays.^153,154^ Additional research is needed using nonconventional biological matrices because they may provide cumulative and/or time varying information on metabolic changes secondary to disease. Further, given that the pharmacokinetics of chemicals favor measurement in different matrices (e.g., hydrophobic chemicals tend to be better measured in plasma, while hydrophilic chemicals are better measured in urine), researchers should consider the use of multiple biomatrix samples in conducting exposomic studies. For example, the use of both plasma and urine would increase overall coverage of the exposome compared to studies that use only a single biomatrix for analysis.

## Precision Exposomics

One of the most important ways to integrate exposomics into healthcare is to focus on precision medicine, which is designed to optimize efficiency, diagnosis, or therapeutic benefit for particular subgroups of patients. To date, precision medicine has focused on genetic or molecular (epigenomics, proteomic, etc.) profiling. However, we know that the environment must be a driver of patient response to treatment or disease progression. For example, if lead is neurotoxic, it stands to reason that exposure to lead will affect the progression of Alzheimer’s Disease or Autism. Further, we are well aware that air pollution affects asthma and that smoking will exacerbate lung and heart disease. Nevertheless, environmental or exposomic issues are rarely considered in precision medicine programs. For precision medicine to be truly precise, there is a clear need to incorporate environmental factors that determine the variability in treatment effects or disease progression. Furthermore, because environmental factors are modifiable, identifying their role in the response to treatment may be actionable. Therefore, a major driver of exposomics should be to identify markers of vulnerability and susceptibility that can be clinically actionable.^155^ To do this, exposomics must expand from public health prevention studies (i.e., case control or longitudinal cohorts of healthy individuals) that address the environmental causes of disease to clinical studies of patients to determine how environment impacts *existing* disease. Although a seemingly subtle shift in focus, the purpose and interpretation of clinical research is very different from public health research. Clinicians may not find causal environmental factors to be useful in patient care. For example, the clinical treatment of a 61-year-old man who has a history of smoking and presents with a lung cancer is not impacted by the smoking history. Decisions on his care, (e.g., surgery, chemotherapy, or even hospice) will be made independent of his smoking history. However, if we begin to measure exposomic chemical signatures when such patients present, we may begin to impact such decisions. It is reasonable to hypothesize that certain signatures predict differential responses to chemotherapeutic agents, resulting in a need to modify their dose to reduce the degree of side effects. Further, as we create better informatic tools to identify the source of chemical signatures (diet, home environment, behavior, geography, cultural practices, water/air quality, etc.), we may be able to alter such signatures toward preferred *treatment* phenotypes. In doing so, we become partners in the growing wave of precision medicine and open the possibility to understand the gene–environment interactions that are impacting clinical outcomes. This information can be combined with data from wearables to provide a digital phenotype that includes a temporal component necessary for establishing causality in relation to environmental exposures.

Exposomic studies combined with wearables may be able to identify previously unknown triggers of disease exacerbation at the individual level, such as triggers of asthma attacks or angina. It would be of particular benefit to identify personalized environmental triggers of disease exacerbation or progression as well as environmental factors that interfere with drug treatment. For example, personal environment sample collecting devices could be carried by individuals with asthma or chronic lung disease.^57^ The collected samples could then be analyzed using mature exposome-based protocols that measure thousands of small molecules and environmental chemicals of concern. A more specific example is that of a metabolomics-based method that monitored real-time exposure to xenobiotics in sweat.^156^ The data provided personalized exposure profiles of individuals that can be coupled to individualized metabolic activities to map the physiological response to exposure. When coupled with a symptom diary or, even better, physiologic data such as that collected by a wearable device,^157^ we can individualize our understanding of the triggers of disease exacerbation. When that is possible, exposomics will then have entered the world of clinical medicine. Further, by doing so, exposomics and environmental health will finally be integrated into medical education, which has been long overdue. As another example, a targeted approach might include screening for the most suspect environmental stressors that have already been linked to a particular disease. Obesogenic chemical exposures such as phthalates could be screened in patients with Type 2 diabetes and linked to measures of insulin resistance (e.g., hemoglobin A1c) and if exposures are associated with higher glucose; then, interventions implemented to reduce phthalate exposure could be made accordingly^158^ with glucose monitoring to establish cause and effect of the intervention. The effect of potential drug–exposome interactions can also be evaluated and catalogued in databases maintained by health care facilities.^159^ Personalized nutrition will be an important component of an exposome-based approach,^160,161^ and dietary intervention remains a readily actionable area. In addition to the medical level, an exposome (i.e., untargeted) approach can also be applied as a public health prevention measure in communities that experience clusters of disease, such as cancer. Such a precision public health initiative could discover chemicals that may impact disease in the community and develop strategies to reduce exposure in susceptible patients. Furthermore, a variety of “omics” platforms can be used to enhance plausibility in the context of environmental health challenges that may be contentious and politically sensitive. Support for mechanistic underpinnings for a charged claim related to social inequities driving adverse health effects, for example, through unbiased observation of DNA methylation associated with various levels of disadvantage, may add value to candidate end point approaches that can be seen as preordained via prior observations. As one specific such application, early life family adversity was shown to be reflected in patterns of DNA methylation in kindergarten children,^162^ increasing the call for interventions to protect youth for such stresses. Given our increasing understanding of the causal role that environmental exposures exert in disease etiology, precision exposomics will become an important component of precision medicine approaches to personalized healthcare as well as public health initiatives.

## Conclusion

The exposome concept is maturing and gaining increasing applications in human and wildlife studies. Significant progress has been made in multiple areas including the following: (1) The scope and composition of the exposome has become clearer. (2) Increased numbers of health researchers have begun including exposomic risks in etiological research. (3) The urgency and importance of funding exposome projects has been acknowledged by governments and funding agencies. (4) Advanced techniques and methods for characterizing exposures and tools for analyzing complex data sets are more available. (5) Databases for compiling and sharing exposome data are evolving quickly. However, there are still multiple obstacles to actionizing the exposome to directly benefit patients and contribute to disease prevention. Some critical challenges include the following: (1) How to untangle the interactions across exposures and those between exposures and genomes, microbiomes, and other endogenous factors that could influence exposure-disease relationships. (2) Replication and validation of the findings will be needed for better delineating the causes of human diseases. (3) Statistical and computational methods will be required to meaningfully associate the exposome to health outcomes. (4) Methods will be needed to characterize personal exposome phenotypes and utilize the information to achieve precision medicine. These efforts will require the combined endeavors of diverse stakeholders including basic scientists, clinicians, policy makers, funders, and the general public to resolve the challenges. Working together across disciplines, we can actionize the exposome to increase our understanding of the etiology of chronic heterogeneous diseases toward the goal of intervention and future disease prevention.

**Key Messages**
(1) The exposome is the interplay of environmental exposure and biological effect. It can be studied top-down, bottom-up, or by an integrated functional approach. Each approach contributes new knowledge and may also lead to sophisticated new hypotheses.
(2) Small molecule profiling by high-resolution mass spectrometry (HRMS) is a key approach for measuring external and internal exposures, including exogenous and endogenous molecules. However, additional efforts to combine GC- and LC-analytical approaches are needed for comprehensive exposomic measures.
(3) Large investments in mass spectrometry infrastructure will be necessary to support human exposome studies on a scale equivalent to the human genome project. This should be done in conjunction with activities in support of method standardization.
(4) Open science workflows and comprehensive public exposome databases in combination with spectral libraries of known and known-unknown substances will accelerate exposome knowledge.
(5) There is a need for significant developments in big data analysis and informatics approaches in order to establish causal links between exposure and adverse health outcomes.
(6) The precision exposome promises to be an important component of precision medicine and precision public health.
(7) Strengthening communication between scientists across disciplines in combination with the development of interdisciplinary exposomics centers is vital for tackling the challenge of the exposome.

## Supporting Information Available

Figure S1: Speaker list for the symposium. Extended discussions of the sections “Considerations in Exposome Study Design” and “Working with Exposome Data”. (PDF)
