# Supplementary material for: Defining the Scope of Exposome Studies and Research Needs from a Multidisciplinary Perspective
Source: Environ Sci Technol Lett. 2021 Sep 7;8(10):839–52. doi: 10.1021/acs.estlett.1c00648 (PMC8515788; doi:10.1021/acs.estlett.1c00648)
Supplement: Supplementary file 1 — ez1c00648_si_001.pdf [file ez1c00648_si_001.pdf]

Supporting Information

Defining the scope of exposome studies and research needs from a multidisciplinary perspective

Pei Zhang[1,2,3], Christopher Carlsten[4], Romanas Chaleckis[1,2], Kati Hanhineva[5,6,7], Mengna Huang[8], Tomohiko Isobe[9], Ville M. Koistinen[5,7], Isabel Meister[1,2], Stefano Papazian[10], Kalliroi Sdougkou[10], Hongyu Xie[10], Jonathan W. Martin[10], Stephen M. Rappaport[11], Hiroshi Tsugawa[12,13,14,15], Douglas I. Walker[16], Tracey J. Woodruff[17], Robert O. Wright[16], and Craig E. Wheelock[1,2,18*]

[1] Gunma University Initiative for Advanced Research (GIAR), Gunma University, Maebashi, Gunma, 371-8511 Japan

[2] Division of Physiological Chemistry 2, Department of Medical Biochemistry and Biophysics, Karolinska Institutet, Stockholm, 171 77 Sweden

[3] Key Laboratory of Drug Quality Control and Pharmacovigilance (Ministry of Education), State Key Laboratory of Natural Medicine, China Pharmaceutical University, Nanjing, 210009 P. R. China

[4] Air Pollution Exposure Laboratory, Division of Respiratory Medicine, Department of Medicine, University of British Columbia, Vancouver, British Columbia, V5Z 1M9 Canada

[5] Department of Life Technologies, Food chemistry and food development unit, University of Turku, Turku, 20014 Finland

[6] Department of Biology and Biological Engineering, Chalmers University of Technology, Gothenburg, SE-412 96 Sweden

[7] Department of Clinical Nutrition and Public Health, University of Eastern Finland, Kuopio, 70210 Finland

[8] Channing Division of Network Medicine, Brigham and Women's Hospital and Harvard Medical School, Boston, Massachusetts, 02115 USA

[9] The Japan Environment and Children's Study Programme Office, National Institute for Environmental Sciences, 16-2 Onogawa, Tsukuba, Ibaraki, 305-8506 Japan

[10] Science for Life Laboratory, Department of Environmental Science, Stockholm University, Stockholm, 114 18 Sweden

[11] Division of Environmental Health Sciences, School of Public Health, University of California, Berkeley, California, 94720-7360 USA

[12] RIKEN Center for Sustainable Resource Science, 1-7-22 Suehiro-cho, Tsurumi-ku, Yokohama, Kanagawa, 230-0045 Japan

[13] RIKEN Center for Integrative Medical Sciences, 1-7-22 Suehiro-cho, Tsurumi-ku, Yokohama, Kanagawa, 230-0045 Japan

[14] Department of Biotechnology and Life Science, Tokyo University of Agriculture and Technology, 2-24-16 Nakamachi, Koganei, Tokyo, 184-8588 Japan

[15] Graduate School of Medical life Science, Yokohama City University, 1-7-22 Suehiro-cho, Tsurumi-ku, Yokohama, 230-0045 Japan

[16] Department of Environmental Medicine and Public Health, Icahn School of Medicine at Mount Sinai, New York, 10029-5674 USA

[17] Program on Reproductive Health and the Environment, University of California San Francisco, San Francisco, California, 94143 USA

[18] Department of Respiratory Medicine and Allergy, Karolinska University Hospital, Stockholm, 141-86 Sweden

* Correspondence to be addressed to:
Craig E. Wheelock, PhD
Email: craig.wheelock@ki.se
Phone: +46 8 524 87630

CONSIDERATIONS IN EXPOSOME STUDY DESIGN

Study design. The first consideration in the design of a top-down or functional exposomic study is to understand the health endpoint under investigation. While the environment can exert a role in nearly all disease processes, the extent of the effect is disease-specific.[1] Most diseases have been categorized in the absence of sufficient exposure-related data, thus exposomics may also contribute to improved phenotyping. While all diseases have environmental contributors, diseases with few established links to the genome may be the most promising candidates for an exposome study, as are diseases occurring in tissues at the interface of exposure (*e.g.,* lungs for respiratory disease,[2-4] skin for dermal disorders,[5-7] gut for gastrointestinal disorders[8-10]).

Most commonly, genetic studies of disease tend to use family-based designs (*i.e.,* enrollment predicated on an affected proband) or unrelated case-controls, where individuals are enrolled after disease diagnosis and matched to healthy controls. These cross-sectional study designs can provide important insight into genetic causes of disease progression and treatment as DNA sequence is static; however, samples collected at study enrollment may not capture past exposure (*e.g.,* early life environmental exposures that contribute to future disease risk[11]). Furthermore, such designs may be subject to reverse causation due to disease-related changes in behavior and biology limiting the causal interpretation of results.

Prospective longitudinal studies that collect exposure samples before disease onset, and ideally in the perinatal period and at several times throughout the life course, have inherent advantages over other study designs that collect exposure sample after enrolling individuals who already have the disease of interest. Large precision medicine studies and other cohorts, such as All of Us in the United States (https://allofus.nih.gov), The Environmental Influences on Child Health Outcomes (ECHO) study in the United States (https://www.nih.gov/research-training/environmental-influences-child-health-outcomes-echo-program),[12] the Trans-Omics for Precision Medicine (TOPMed) program (https://www.nhlbiwgs.org), and the European Prospective Investigation of Cancer (EPIC) (https://epic.iarc.fr), provide key opportunities for studying how the exposome contributes to disease risk before diagnosis. Other designs include case studies, cohort studies, case-control studies, nested case-control studies, and cross-sectional studies. While there is a risk of reverse causation in case-control study designs, these may provide the only realistic option for rare diseases. In large and well-resourced cohort studies, the prospective nested case-control study design becomes feasible. A notable exception to the need for prospective

longitudinal exposure data is when exposure can be accurately evaluated retrospectively, for example for external exposures using air pollution and satellite records, or when the exposure is stable over time and simple to retrieve in an unbiased fashion from public databases (*e.g.,* built environment[13]). Innovative new assays may also provide a retrospective record of exposure, such as through the tooth exposome.[14, 15]

It is not currently feasible to acquire data on all possible exposures in a given study. It is therefore natural to narrow the focus in exposome studies (*e.g.,* small molecules in biofluids or in air pollution). Given limited resources, gathering high-quality information on biological responses or health outcomes (both negative and positive) is of equal importance in exploratory studies and may enable deeper understanding of mechanisms of disease progression following exposure. Individual meta-data, patient demographics, electronic medical records, and public databases should all be utilized for this purpose with due consideration to ethics and general data protection regulations.

Population sample size. An inherent challenge of exposomics is that exposome studies will need to be larger than studies in which only a single chemical is tested. Alternatively, smaller studies might be combined into statistical evaluations; however, this will require standardization of the exposure and health measurements. The study size is typically predetermined by the need to achieve a particular statistical power for a targeted effect size using estimates of the dose response slope; however, it is unclear as to what effect sizes can be expected in exposome studies, particularly if the effects are not confined to one particular disease or one particular exposure. Identifying appropriate levels of power for exposome studies is further complicated due to the large number of detected signals and need to correct for multiple testing, correlation among co-exposures, measurement error among exposome features, and variable detection rates based on age, location and other covariates.[16] One strategy is to leverage previous exposome studies, which are often underpowered but can provide an estimate on data correlation structure and effect examples. For example, Jung *et al* leveraged an exposome-wide association study (ExWAS) framework that combined measurement of 128 endocrine disrupting compounds with a population size of 473 men to investigate environmental impacts on semen quality parameters.[17] After correcting for multiple testing, none of the exposures reached significance and post-hoc statistical analysis suggested a mean sample size of 2,696 participants was required to achieve power >0.8. This number is significantly higher than published studies to date and highlights the importance of evaluating power both prior to and after statistical analysis when interpreting results.

However, this study did not consider mixture effects or latent structures within the data, which are key to understanding relationships between the exposome and adverse outcomes. Current evidence suggests it is the joint effect of multiple, low-level exposures driving disease risk rather than a small number of independent exposures.[18] Comparison of linear-regression and other statistical analysis approaches for variable selection demonstrate that ExWAS based upon individual linear regression with correction for multiple testing evidences the poorest performance when compared to other strategies due to the complex correlation structure of exposome data.[19, 20] Reported exposome-based studies to date vary widely in size, which is at least partly related to study aim; however, large population sizes are critical due to the widespread variability in exposure patterns across a population that can be influenced by many different factors.

The Japan Environment and Children's Study (JECS) recruited approximately 100,000 birth-pairs and 50,000 fathers, with multiple priority outcomes including reproduction/pregnancy complications, congenital anomalies, neuropsychiatric disorders, immune system disorders, and metabolic/endocrine system disorders.[21] The human early-life exposome (HELIX) project included 1301 birth-pairs from six European birth cohorts and plans to study the role of exposome on childhood obesity, cardiometabolic risk, blood pressure abnormalities, neurodevelopment problems, and respiratory diseases (asthma, wheeze, lung function).[22] Conversely, a longitudinal exposome study recruited only 15 individuals who were followed for up to two years.[23] However, this study eventually obtained 70 billion readouts and showed in great detail the tremendous dynamics and diversity of the exposome. Controlled human exposure studies are also of modest size, but given intra-individual control (greatly reducing confounding), significant changes in outcome can be fairly attributed to the variable exposure. For example, using proteomics, such a study was able to profile the difference in airway protein response to different combinations of inhalants, supporting 'omics as a way to distinguish between exposure combinations and, in particular, showing that adding diesel exhaust to allergen altered host defense.[24, 25] Thus, sample size in exposome studies accordingly depends on the study design, desired endpoint, rate of the disease occurrence (in prospective studies) and available resources.

Windows of susceptibility. The timing of sample collection is important and can depend on the health outcomes of interest. Windows of susceptibility are the life periods in which an organism may be most sensitive to exposures. If an exposure occurs in such a window, the disease may progress quickly or may appear decades later.[26-28] Without prior knowledge,

longitudinal sampling is essential to ensure having samples to measure exposure during periods of heightened susceptibility. To truly sequence the exposome, defined as throughout the entire life-course, sampling should technically begin early in a pregnancy, or even prior to conception. In fact, a general hypothesis grounded in epidemiological evidence is that health and disease is largely dependent on exposure early in life, including during fetal stages (*i.e.,* DOHaD Hypothesis[29]). Because exposure windows likely deviate by age and health outcomes, defining the "average" exposome by age and disease status will be key measures for understanding how the human exposome contributes to disease risk; concepts being examined by the ECHO program as well as recent EU projects (*e.g.,* https://longitools.org/).

Replication and validation. Top-down and bottom-up exposome studies in human populations will be hypothesis generating, and should eventually lead to more sophisticated and larger functional exposomic study designs where associations between exposure and effect are hypothesis tested. Establishing causal links will require a cautious approach, as false positives are likely even after stringent quality control, data pre-processing, and data analysis. While false positive rates will be high in any omics study, false negatives can also arise from either low sensitivity of the analytical methods or unexpectedly low exposure rates in the study population. For this reason, all resources cannot be placed into a single human exposome study, such studies must be replicated and the results reproduced for validation, including in longitudinal and mechanistic studies in subcohorts of the same populations. Validation needs to be performed in an independent sample set, demonstrating that the findings are replicable beyond the initial study population.[30] A particular challenge of validating an exposome study is that exposures will vary across populations, rendering it necessary to have precise information on the exposures in order to establish the exposure-disease relationship. Some associations can be tested for biological plausibility using controlled laboratory experiments, for example by combining chemical exposures with other environmental stresses and monitoring pathophysiology using *in vitro* or *in vivo* models.[31] However, this approach will require the study to be designed appropriately with similar exposure levels administered during the appropriate window(s) of susceptibility. Hence, epidemiologic data may need to precede such studies in order to inform dose, dose combinations and timing of exposure.

Biological sampling. Blood plasma or serum are the most common matrices in human studies of organic contaminant exposures; however, whole blood (or the red blood cell fraction) offers the possibility for the measurement of metals, trace elements, and methyl

mercury, etc.[32-34] Interestingly, some per- and polyfluoroalkyl substances (PFASs) have also been found to partition preferentially in the red blood cell fraction.[35] The red blood cell fraction is also optimal for protein adductomics, because the lack of a nucleus in red blood cells means that covalent reactions leave a lasting imprint (*i.e.,* no *de novo* protein synthesis occurs). Hemoglobin adducts for a list of reactive chemicals have been summarized [36] and proposed as unbiased measures of total exposure to reactive electrophiles.[37, 38] Thus, for a comprehensive exposome study, whole blood, or separated fractions of plasma/serum and blood cells should be considered. These same samples can often be used for diagnosis of disease or biological effects, for example plasma and serum are commonly used for metabolomics, proteomics, and transcriptomics, while the epigenome can be evaluated in leukocytes.

Another useful matrix is urine, which is a more easily accessible and less invasive to collect than blood, and moreover can be collected by the participant outside of the clinic. In addition, urine is clearly a useful matrix for studies of renal disease. Other potential matrices that can answer specific questions include feces, teeth, hair, saliva, tears, skin and even earwax. Tissue samples, such as placenta or biopsies may, depending on the research question, be a useful matrix. There are advantages to analyzing a matrix for both bottom-up and top-down approaches that is most closely related to the disease. For example, in respiratory disease, the analysis of lung fluid, breath condensate, or sputum is preferred given that these matrices are closest to the target organ of the disease.[2-4] Saliva may be of value when studying oral health, and fecal samples may help in assessing the causes of gut-related symptoms[8-10]. These matrices can be more challenging and expensive to obtain, process, normalize and store than more common fluids such as blood and urine; however, obtaining multiple matrices from the same individual may increase understanding of the internal exposome.

WORKING WITH EXPOSOME DATA

Analyzing, interpreting, and reporting exposomic data. Understanding the link between the exposome and health outcomes is a challenge, and currently there are only a few approaches applied in large scale exposome studies.[39] The most commonly used method is the exposome-wide association study, which is an exposure-by-exposure method and consists of a covariate-by-covariate estimation of the exposure outcome association through

independent linear regression models. While it is useful for hypothesis generation, this approach does not implicitly consider correlations between exposures and tends to give a high rate of false positives when insufficiently corrected for multiple comparisons.[20] A recent R package called rexposome is designed for the analysis of exposome data and offers a set of functions to incorporate exposome data into the R framework Biocondcutor. Borrowing from other omics, for mechanistic inference, we may employ data-driven network methods such as weighted correlation network analysis[40] and Gaussian graphical models.[41, 42] If a widely-accepted exposome database can be established, knowledge-driven approaches such as pathway enrichment analysis[43] may also be applied. When prediction of health outcomes is the goal, approaches including penalized regression methods (*e.g.,* elastic net[44]), partial least squares discriminant analysis and its variations,[45] and conditional Gaussian Bayesian networks[46] may be considered. Exposomic data could be integrated with other omics data using causal mediation approaches,[47] Mendelian randomization,[48] joint effect hypothesis testing,[49] and similarity network fusion,[50] etc., if existing evidence alludes to possible mediation/interaction.

An explicit agreement on data acquisition strategies may facilitate or optimize data analysis strategies. However, it is not realistic to expect the academic community to coalesce around a given method, and bioinformatic methods continue to advance remarkably such that data captured in even unanticipated ways can be meaningfully and effectively synthesized and distilled by evolving informatic techniques, including machine learning and artificial intelligence (AI). Therefore, for some studies it will remain necessary to customize analytical approaches while for others this presumed need may prove less restrictive. Even if the promise of new analytics, including AI, is widely achieved, a detailed reporting of method parameters in both quality control and data analysis will likely be necessary. Accordingly, investigators should report the history of data processing used to obtain the final results, including detailed software parameter settings, in addition to making the raw data accessible. For example, in HRMS-based exposomics, the MS raw data should be deposited in repositories along with experimental metadata (*e.g.,* GNPS MassIVE, Metabolomics Workbench, Metabolights) and if there is any programming applied, the source code should be deposited in code sharing platforms like GitHub (https://github.com/). In addition, more detailed experimental methods could be readily shared using open access tools such as Jupyter notebooks (https://jupyter.org).

A particular weak link in analyzing exposomics data is in meta-analysis, which has been used extensively for data from targeted studies[51, 52] but not from untargeted studies. There is need to increase the crosstalk between the different stakeholders from epidemiologists to laboratory scientists. By strengthening the communication among the diverse scientists working in the exposome space, we can begin to develop the tools and approaches necessary to perform the large-scale data analysis required of true exposomics. In particular, advances in predictive analytics and inferential statistics will be required to integrate the multiple data modalities needed to understand the exposome and apply it within a precision public health context,[53, 54] which will necessitate the advent of additional exposome ontologies.[55]

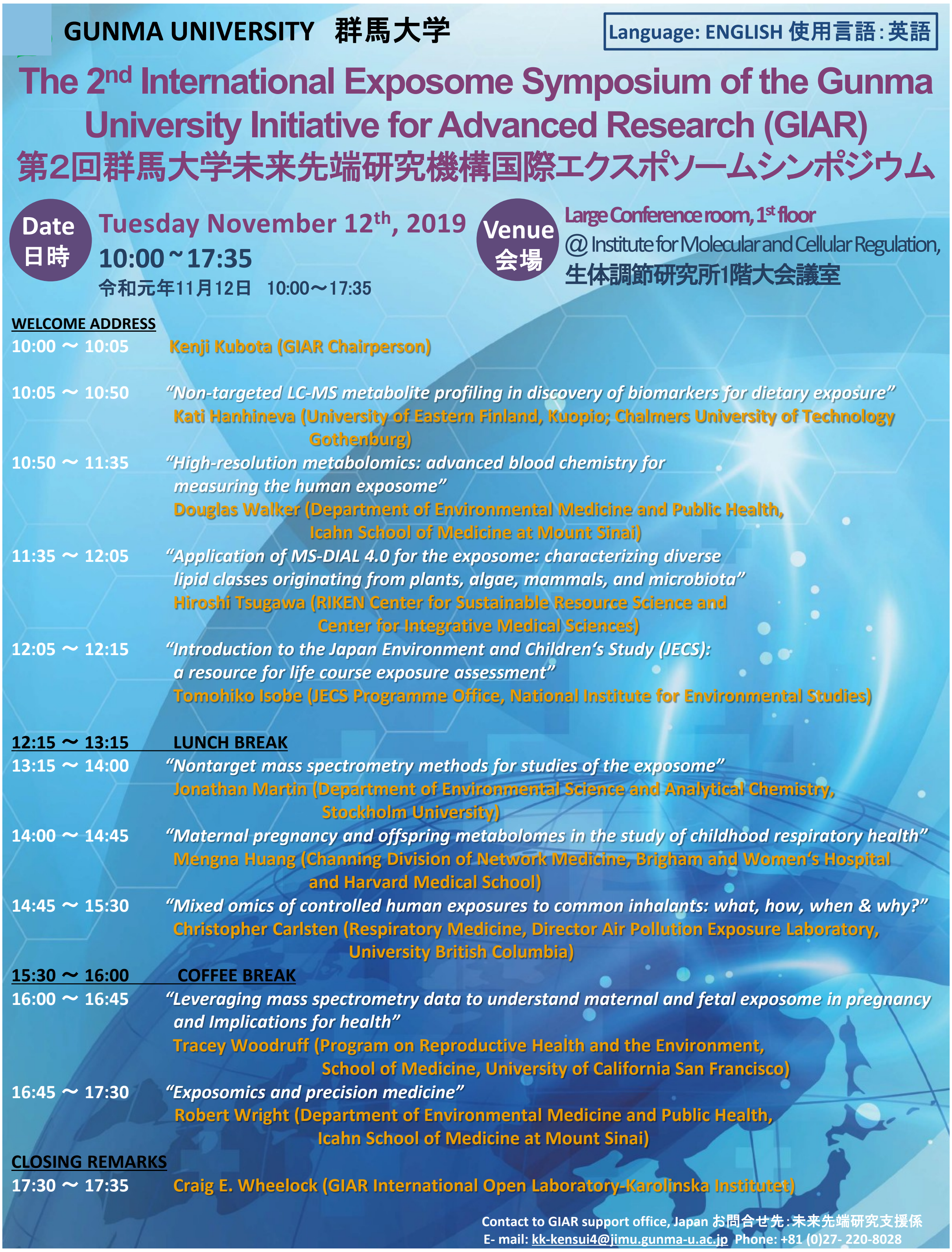

Figure S1. Program and speaker list for the Gunma University Initiative for Advanced Research (GIAR) exposome workshop.

References

1. Rappaport, S. M., Genetic Factors Are Not the Major Causes of Chronic Diseases. *PLoS One* **2016,** *11*, (4), e0154387.
2. Burbank, A. J.; Sood, A. K.; Kesic, M. J.; Peden, D. B.; Hernandez, M. L., Environmental determinants of allergy and asthma in early life. *J Allergy Clin Immunol* **2017,** *140*, (1), 1-12.
3. Wheelock, C. E.; Rappaport, S. M., The role of gene-environment interactions in lung disease: the urgent need for the exposome. *Eur Respir J* **2020,** *55*, (2).
4. Guillien, A.; Cadiou, S.; Slama, R.; Siroux, V., The Exposome Approach to Decipher the Role of Multiple Environmental and Lifestyle Determinants in Asthma. *Int J Environ Res Public Health* **2021,** *18*, (3).
5. Celebi Sozener, Z.; Cevhertas, L.; Nadeau, K.; Akdis, M.; Akdis, C. A., Environmental factors in epithelial barrier dysfunction. *J Allergy Clin Immunol* **2020,** *145*, (6), 1517-1528.
6. Cecchi, L.; D'Amato, G.; Annesi-Maesano, I., External exposome and allergic respiratory and skin diseases. *J Allergy Clin Immunol* **2018,** *141*, (3), 846-857.
7. Appenzeller, B. M. R.; Chadeau-Hyam, M.; Aguilar, L., Skin exposome science in practice : current evidence on hair biomonitoring and future perspectives. *J Eur Acad Dermatol Venereol* **2020,** *34 Suppl 4*, 26-30.
8. Sbihi, H.; Boutin, R. C.; Cutler, C.; Suen, M.; Finlay, B. B.; Turvey, S. E., Thinking bigger: How early-life environmental exposures shape the gut microbiome and influence the development of asthma and allergic disease. *Allergy* **2019,** *74*, (11), 2103-2115.
9. Sinisalu, L.; Sen, P.; Salihovic, S.; Virtanen, S. M.; Hyoty, H.; Ilonen, J.; Toppari, J.; Veijola, R.; Oresic, M.; Knip, M.; Hyotylainen, T., Early-life exposure to perfluorinated alkyl substances modulates lipid metabolism in progression to celiac disease. *Environ Res* **2020,** *188*, 109864.
10. Pimentel, M.; Lembo, A., Microbiome and Its Role in Irritable Bowel Syndrome. *Dig Dis Sci* **2020,** *65*, (3), 829-839.
11. Heindel, J. J.; Vandenberg, L. N., Developmental origins of health and disease: a paradigm for understanding disease cause and prevention. *Curr Opin Pediatr* **2015,** *27*, (2), 248-53.
12. Volk, H. E.; Perera, F.; Braun, J. M.; Kingsley, S. L.; Gray, K.; Buckley, J.; Clougherty, J. E.; Croen, L. A.; Eskenazi, B.; Herting, M.; Just, A. C.; Kloog, I.; Margolis, A.; McClure, L. A.; Miller, R.; Levine, S.; Wright, R.; Environmental influences on Child Health, O., Prenatal air pollution exposure and neurodevelopment: A review and blueprint for a harmonized approach within ECHO. *Environ Res* **2020**, 110320.
13. Dai, D.; Prussin, A. J., 2nd; Marr, L. C.; Vikesland, P. J.; Edwards, M. A.; Pruden, A., Factors Shaping the Human Exposome in the Built Environment: Opportunities for Engineering Control. *Environ Sci Technol* **2017,** *51*, (14), 7759-7774.
14. Andra, S. S.; Austin, C.; Arora, M., The tooth exposome in children's health research. *Curr Opin Pediatr* **2016,** *28*, (2), 221-7.

15. Arora, M.; Austin, C., Teeth as a biomarker of past chemical exposure. *Curr Opin Pediatr* **2013,** *25*, (2), 261-7.

16. Patel, C. J., Analytic Complexity and Challenges in Identifying Mixtures of Exposures Associated with Phenotypes in the Exposome Era. *Curr Epidemiol Rep* **2017,** *4*, (1), 22-30.

17. Chung, M. K.; Buck Louis, G. M.; Kannan, K.; Patel, C. J., Exposome-wide association study of semen quality: Systematic discovery of endocrine disrupting chemical biomarkers in fertility require large sample sizes. *Environ Int* **2019,** *125*, 505-514.

18. Tanner, E. M.; Bornehag, C. G.; Gennings, C., Repeated holdout validation for weighted quantile sum regression. *MethodsX* **2019,** *6*, 2855-2860.

19. Barrera-Gomez, J.; Agier, L.; Portengen, L.; Chadeau-Hyam, M.; Giorgis-Allemand, L.; Siroux, V.; Robinson, O.; Vlaanderen, J.; Gonzalez, J. R.; Nieuwenhuijsen, M.; Vineis, P.; Vrijheid, M.; Vermeulen, R.; Slama, R.; Basagana, X., A systematic comparison of statistical methods to detect interactions in exposome-health associations. *Environ Health* **2017,** *16*, (1), 74.

20. Agier, L.; Portengen, L.; Chadeau-Hyam, M.; Basagana, X.; Giorgis-Allemand, L.; Siroux, V.; Robinson, O.; Vlaanderen, J.; Gonzalez, J. R.; Nieuwenhuijsen, M. J.; Vineis, P.; Vrijheid, M.; Slama, R.; Vermeulen, R., A Systematic Comparison of Linear Regression-Based Statistical Methods to Assess Exposome-Health Associations. *Environ Health Perspect* **2016,** *124*, (12), 1848-1856.

21. Kawamoto, T.; Nitta, H.; Murata, K.; Toda, E.; Tsukamoto, N.; Hasegawa, M.; Yamagata, Z.; Kayama, F.; Kishi, R.; Ohya, Y.; Saito, H.; Sago, H.; Okuyama, M.; Ogata, T.; Yokoya, S.; Koresawa, Y.; Shibata, Y.; Nakayama, S.; Michikawa, T.; Takeuchi, A.*, et al.*, Rationale and study design of the Japan environment and children's study (JECS). *BMC Public Health* **2014,** *14*, 25.

22. Vrijheid, M.; Slama, R.; Robinson, O.; Chatzi, L.; Coen, M.; van den Hazel, P.; Thomsen, C.; Wright, J.; Athersuch, T. J.; Avellana, N.; Basagana, X.; Brochot, C.; Bucchini, L.; Bustamante, M.; Carracedo, A.; Casas, M.; Estivill, X.; Fairley, L.; van Gent, D.; Gonzalez, J. R.*, et al.*, The human early-life exposome (HELIX): project rationale and design. *Environ Health Perspect* **2014,** *122*, (6), 535-44.

23. Jiang, C.; Wang, X.; Li, X.; Inlora, J.; Wang, T.; Liu, Q.; Snyder, M., Dynamic Human Environmental Exposome Revealed by Longitudinal Personal Monitoring. *Cell* **2018,** *175*, (1), 277-291 e31.

24. Piyadasa, H.; Hemshekhar, M.; Carlsten, C.; Mookherjee, N., Inhaled Diesel Exhaust Decreases the Antimicrobial Peptides alpha-Defensin and S100A7 in Human Bronchial Secretions. *Am J Respir Crit Care Med* **2018,** *197*, (10), 1358-1361.

25. Mookherjee, N.; Piyadasa, H.; Ryu, M. H.; Rider, C. F.; Ezzati, P.; Spicer, V.; Carlsten, C., Inhaled diesel exhaust alters the allergen-induced bronchial secretome in humans. *Eur Respir J* **2018,** *51*, (1).

26. Xu, K.; An, N.; Huang, H.; Duan, L.; Ma, J.; Ding, J.; He, T.; Zhu, J.; Li, Z.; Cheng, X.; Zhou, G.; Ba, Y., Fluoride exposure and intelligence in school-age children: evidence from different windows of exposure susceptibility. *BMC Public Health* **2020,** *20*, (1), 1657.

27. Bose, S.; Ross, K. R.; Rosa, M. J.; Chiu, Y. M.; Just, A.; Kloog, I.; Wilson, A.; Thompson, J.; Svensson, K.; Rojo, M. M. T.; Schnaas, L.; Osorio-Valencia, E.; Oken, E.; Wright, R. O.; Wright, R. J., Prenatal particulate air pollution exposure and sleep disruption in preschoolers: Windows of susceptibility. *Environ Int* **2019,** *124*, 329-335.

28. Wang, Q.; Benmarhnia, T.; Zhang, H.; Knibbs, L. D.; Sheridan, P.; Li, C.; Bao, J.; Ren, M.; Wang, S.; He, Y.; Zhang, Y.; Zhao, Q.; Huang, C., Identifying windows of susceptibility for maternal exposure to ambient air pollution and preterm birth. *Environ Int* **2018,** *121*, (Pt 1), 317-324.

29. Gillman, M. W., Developmental origins of health and disease. *N Engl J Med* **2005,** *353*, (17), 1848-50.

30. Igl, B. W.; Konig, I. R.; Ziegler, A., What do we mean by 'replication' and 'validation' in genome-wide association studies? *Hum Hered* **2009,** *67*, (1), 66-8.

31. Antonini, J. M.; Kodali, V.; Shoeb, M.; Kashon, M.; Roach, K. A.; Boyce, G.; Meighan, T.; Stone, S.; McKinney, W.; Boots, T.; Roberts, J. R.; Zeidler-Erdely, P. C.; Erdely, A., Effect of a High-Fat Diet and Occupational Exposure in Different Rat Strains on Lung and Systemic Responses: Examination of the Exposome in an Animal Model. *Toxicol Sci* **2020,** *174*, (1), 100-111.

32. Wiseman, C. L. S.; Parnia, A.; Chakravartty, D.; Archbold, J.; Copes, R.; Cole, D., Total, methyl and inorganic mercury concentrations in blood and environmental exposure sources in newcomer women in Toronto, Canada. *Environ Res* **2019,** *169*, 261-271.

33. Wojciak-Kosior, M.; Szwerc, W.; Strzemski, M.; Wichlacz, Z.; Sawicki, J.; Kocjan, R.; Latalski, M.; Sowa, I., Optimization of high-resolution continuum source graphite furnace atomic absorption spectrometry for direct analysis of selected trace elements in whole blood samples. *Talanta* **2017,** *165*, 351-356.

34. Gil, F.; Hernandez, A. F.; Marquez, C.; Femia, P.; Olmedo, P.; Lopez-Guarnido, O.; Pla, A., Biomonitorization of cadmium, chromium, manganese, nickel and lead in whole blood, urine, axillary hair and saliva in an occupationally exposed population. *Sci Total Environ* **2011,** *409*, (6), 1172-80.

35. Jin, H.; Zhang, Y.; Jiang, W.; Zhu, L.; Martin, J. W., Isomer-Specific Distribution of Perfluoroalkyl Substances in Blood. *Environ Sci Technol* **2016,** *50*, (14), 7808-15.

36. Rubino, F. M.; Pitton, M.; Di Fabio, D.; Colombi, A., Toward an "omic" physiopathology of reactive chemicals: thirty years of mass spectrometric study of the protein adducts with endogenous and xenobiotic compounds. *Mass Spectrom Rev* **2009,** *28*, (5), 725-84.

37. Golime, R.; Chandra, B.; Palit, M.; Dubey, D. K., Adductomics: a promising tool for the verification of chemical warfare agents' exposures in biological samples. *Arch Toxicol* **2019,** *93*, (6), 1473-1484.

38. Shibata, T.; Uchida, K., Protein adductomics: A comprehensive analysis of protein modifications by electrophiles. *Free Radic Biol Med* **2019,** *144*, 218-222.

39. Santos, S.; Maitre, L.; Warembourg, C.; Agier, L.; Richiardi, L.; Basagana, X.; Vrijheid, M., Applying the exposome concept in birth cohort research: a review of statistical approaches. *Eur J Epidemiol* **2020,** *35*, (3), 193-204.

40. Langfelder, P.; Horvath, S., WGCNA: an R package for weighted correlation network analysis. *BMC Bioinform* **2008,** *9*, 559.

41. Krumsiek, J.; Suhre, K.; Illig, T.; Adamski, J.; Theis, F. J., Gaussian graphical modeling reconstructs pathway reactions from high-throughput metabolomics data. *Bmc Syst Biol* **2011,** *5*, 21.

42. Krumsiek, J.; Suhre, K.; Evans, A. M.; Mitchell, M. W.; Mohney, R. P.; Milburn, M. V.; Wagele, B.; Romisch-Margl, W.; Illig, T.; Adamski, J.; Gieger, C.; Theis, F. J.; Kastenmuller, G., Mining the unknown: a systems approach to metabolite identification combining genetic and metabolic information. *PLoS Genet* **2012,** *8*, (10), e1003005.

43. Subramanian, A.; Tamayo, P.; Mootha, V. K.; Mukherjee, S.; Ebert, B. L.; Gillette, M. A.; Paulovich, A.; Pomeroy, S. L.; Golub, T. R.; Lander, E. S.; Mesirov, J. P., Gene set enrichment analysis: a knowledge-based approach for interpreting genome-wide expression profiles. *Proc Natl Acad Sci U S A* **2005,** *102*, (43), 15545-50.

44. Zou, H.; Hastie, T., Regularization and variable selection via the elastic net. *J R Stat Soc Series B Stat Methodol* **2005,** *67*, (2), 301-320.

45. Le Cao, K. A.; Boitard, S.; Besse, P., Sparse PLS discriminant analysis: biologically relevant feature selection and graphical displays for multiclass problems. *BMC Bioinform* **2011,** *12*, 253.

46. McGeachie, M. J.; Chang, H. H.; Weiss, S. T., CGBayesNets: conditional Gaussian Bayesian network learning and inference with mixed discrete and continuous data. *PLoS Comput Biol* **2014,** *10*, (6), e1003676.

47. VanderWeele, T. J.; Vansteelandt, S., Mediation Analysis with Multiple Mediators. *Epidemiol Methods* **2014,** *2*, (1), 95-115.

48. Davey Smith, G.; Hemani, G., Mendelian randomization: genetic anchors for causal inference in epidemiological studies. *Hum Mol Genet* **2014,** *23*, (R1), R89-98.

49. Chu, S. H.; Huang, Y. T., Integrated genomic analysis of biological gene sets with applications in lung cancer prognosis. *BMC Bioinform* **2017,** *18*, (1), 336.

50. Wang, B.; Mezlini, A. M.; Demir, F.; Fiume, M.; Tu, Z.; Brudno, M.; Haibe-Kains, B.; Goldenberg, A., Similarity network fusion for aggregating data types on a genomic scale. *Nat Methods* **2014,** *11*, (3), 333-7.

51. Robinson, O.; Tamayo, I.; de Castro, M.; Valentin, A.; Giorgis-Allemand, L.; Hjertager Krog, N.; Marit Aasvang, G.; Ambros, A.; Ballester, F.; Bird, P.; Chatzi, L.; Cirach, M.; Dedele, A.; Donaire-Gonzalez, D.; Grazuleviciene, R.; Iakovidis, M.; Ibarluzea, J.; Kampouri, M.; Lepeule, J.; Maitre, L.*, et al.*, The Urban Exposome during Pregnancy and Its Socioeconomic Determinants. *Environ Health Perspect* **2018,** *126*, (7), 077005.

52. Fiolet, T.; Mahamat-Saleh, Y.; Frenoy, P.; Kvaskoff, M.; Romana Mancini, F., Background exposure to polychlorinated biphenyls and all-cause, cancer-specific, and cardiovascular-specific mortality: A systematic review and meta-analysis. *Environ Int* **2021,** *154*, 106663.

53. Manrai, A. K.; Cui, Y.; Bushel, P. R.; Hall, M.; Karakitsios, S.; Mattingly, C. J.; Ritchie, M.; Schmitt, C.; Sarigiannis, D. A.; Thomas, D. C.; Wishart, D.; Balshaw, D. M.; Patel, C. J., Informatics and Data Analytics to Support Exposome-Based Discovery for Public Health. *Annu Rev Public Health* **2017,** *38*, 279-294.

54. Pearson, T. A.; Califf, R. M.; Roper, R.; Engelgau, M. M.; Khoury, M. J.; Alcantara, C.; Blakely, C.; Boyce, C. A.; Brown, M.; Croxton, T. L.; Fenton, K.; Green Parker, M. C.; Hamilton, A.; Helmchen, L.; Hsu, L. L.; Kent, D. M.; Kind, A.; Kravitz, J.; Papanicolaou, G. J.; Prosperi, M.*, et al.*, Precision Health Analytics With Predictive Analytics and Implementation Research: JACC State-of-the-Art Review. *J Am Coll Cardiol* **2020,** *76*, (3), 306-320.

55. Zhang, H.; Hu, H.; Diller, M.; Hogan, W. R.; Prosperi, M.; Guo, Y.; Bian, J., Semantic standards of external exposome data. *Environ Res* **2021,** *197*, 111185.
